# Supplementary material for: Differential Labeling of Glycoproteins with Alkynyl Fucose Analogs
Source: Int J Mol Sci. 2020 Aug 20;21(17):6007. doi: 10.3390/ijms21176007 (PMC7503990; doi:10.3390/ijms21176007)
Supplement: Supplementary file 1 [file ijms-21-06007-s001.pdf]

Supplementary Figure 1

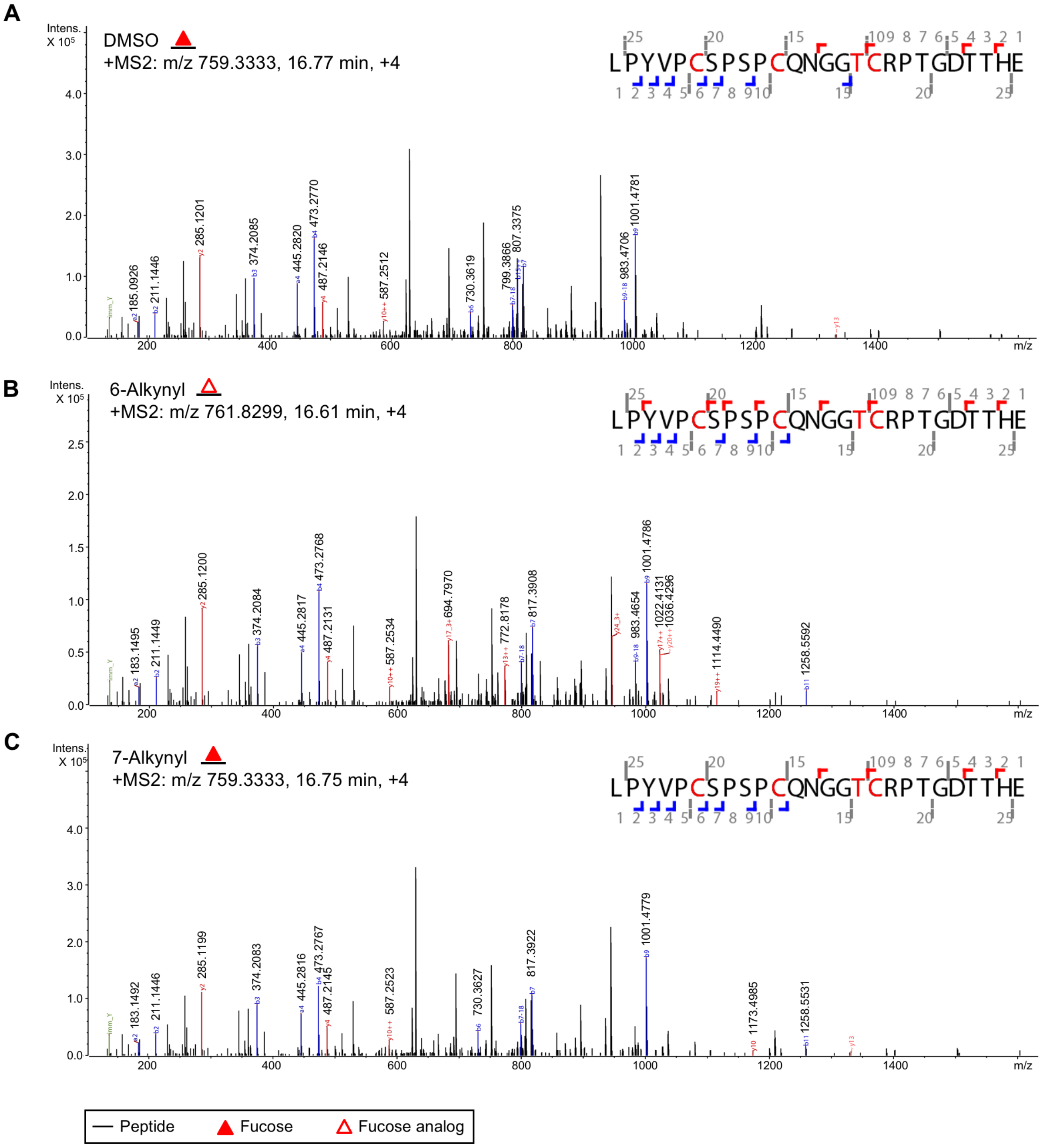

Supplemental Figure 1. Mass spectra corresponding to EICs in Figure 2D. MS2 spectra of glycoforms of the peptide LPYVPCSPSPCQNGGTCRPTGDTTHE from mNOTCH1 EGF6 confirm the peptide assignment. (**A**) MS2 spectra of the peptide modified with Fuc in sample treated with DMSO. (**B**) MS2 spectra of the peptide modified with 6-Alk-Fuc in sample treated with peracetylated 6-Alk-Fuc. (**C**) MS2 spectra of the peptide modified with Fuc in sample treated with peracetylated 7-Alk-Fuc.

Supplementary Figure 2

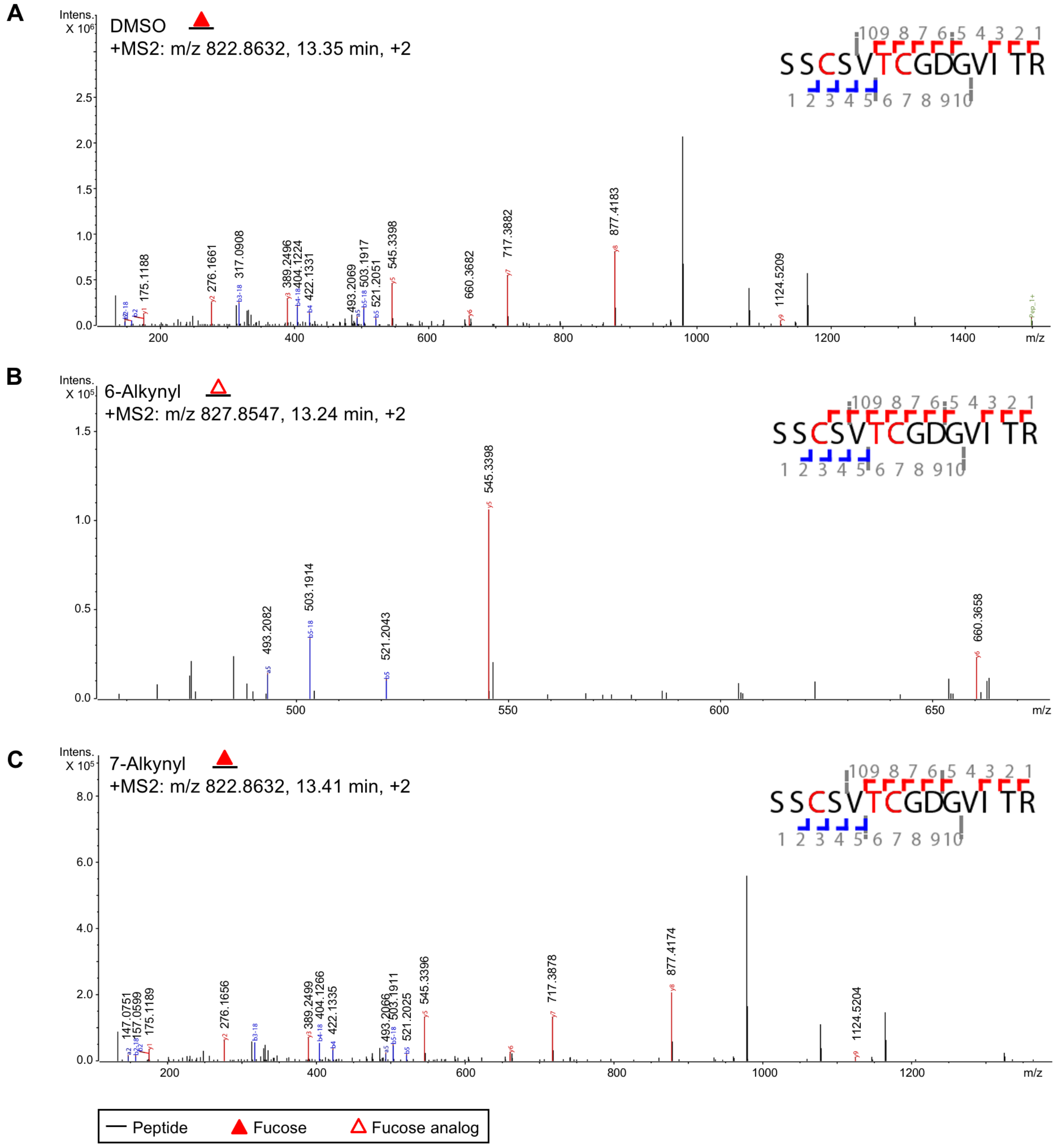

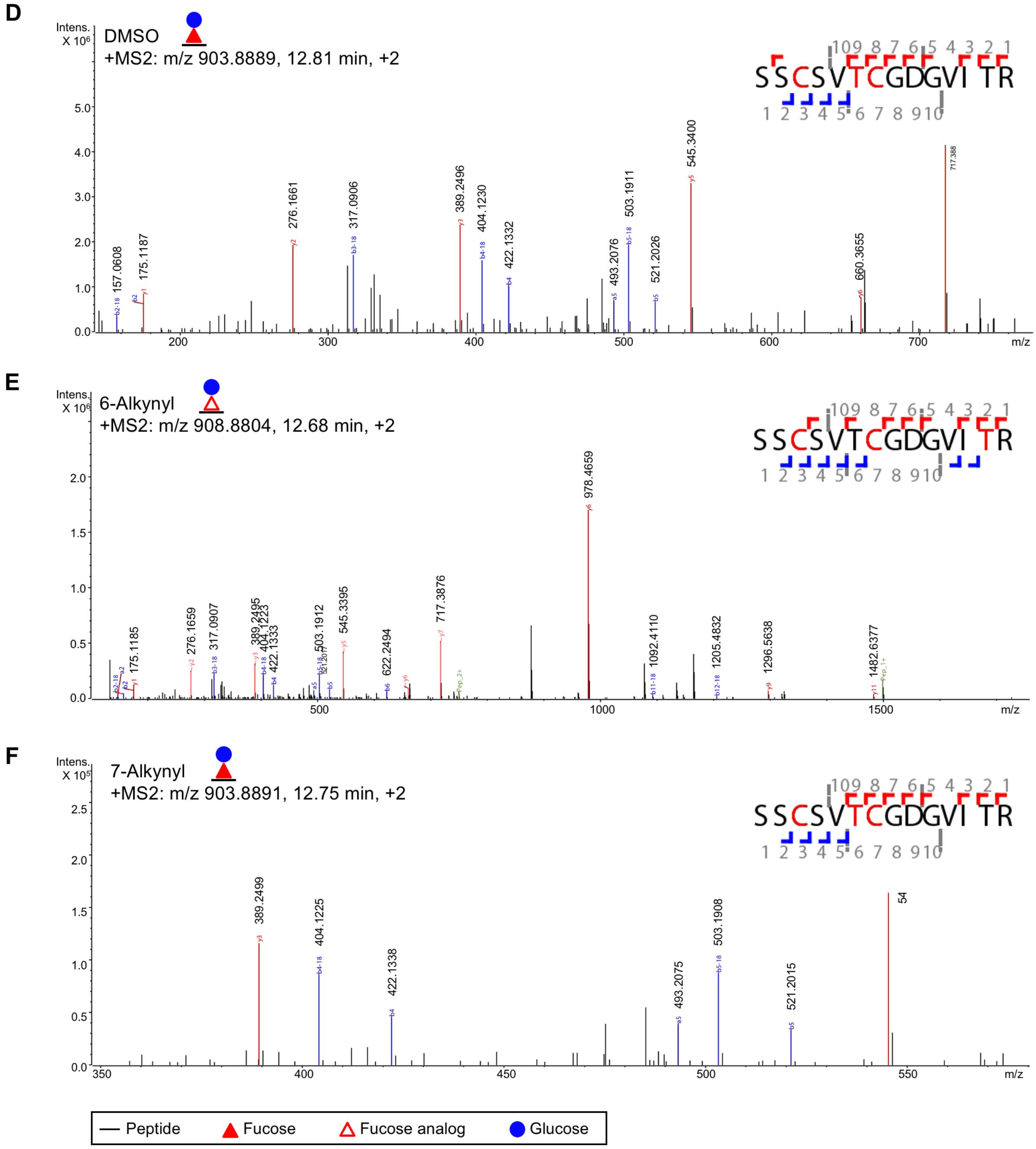

Supplemental Figure 2. Mass spectra corresponding to EICs in Figure 2F. MS2 spectra of glycoforms of the peptide SSCSVTCGDGVITR from hTHBS1 TSR2 confirm the peptide assignment. (**A**) MS2 spectra of the peptide modified with Fuc monosaccharide in sample treated with DMSO. (**B**) MS2 of the peptide modified with 6-Alk-Fuc monosaccharide in sample treated with peracetylated 6-Alk-Fuc. (**C**) MS2 spectra of the peptide modified with Fuc monosaccharide in sample treated with peracetylated 7-Alk-fuc. (**D**) MS2 spectra of the peptide modified with Glc-Fuc disaccharide in sample treated with DMSO. (**E**) MS2 of the peptide modified with Glc-6-Alk-Fuc disaccharide in sample treated with peracetylated 6-Alk-Fuc. (**F**) MS2 spectra of the peptide modified with Glc-Fuc disaccharide in sample treated with peracetylated 7-Alk-Fuc.

Supplementary Figure 3

hTHBS1 TSR1-3
TSR 3
DICSVTCGGGVQK

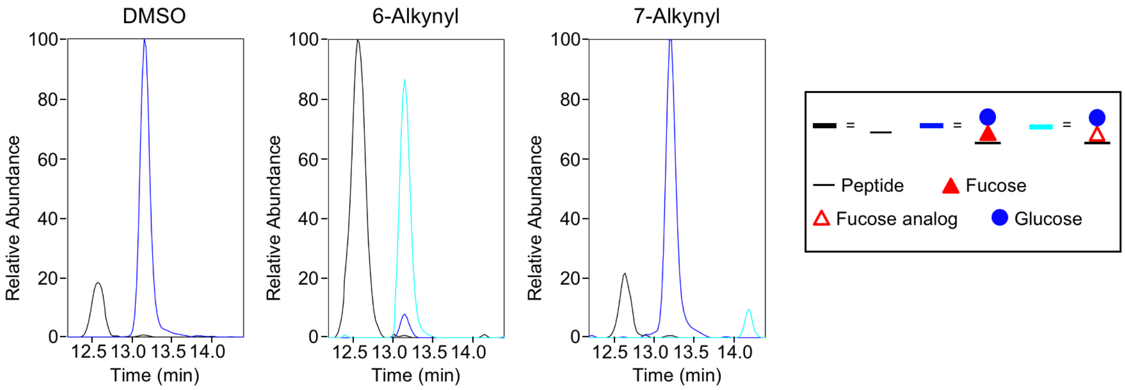

Supplemental Figure 3. 6-Alk-Fuc but not 7-Alk-Fuc is incorporated into TSR3 of hTHBS1 TSR1-3. HEK293T cells were transfected with hTHBS1 TSR1-3 plasmid and incubated with 50 μM peracetylated 6-Alk-Fuc or 7-Alk-Fuc for 3 days. hTHBS1 TSR1-3 was purified from medium and analyzed by mass spectrometry as described in Methods. An EIC of the different glycoforms of a peptide from hTHBS1 TSR3 was prepared. Black line, unmodified; blue line, Glc-Fuc modified; aqua line, Glc-6-Alk-Fuc or Glc-7-Alk-Fuc.

Supplementary Figure 4

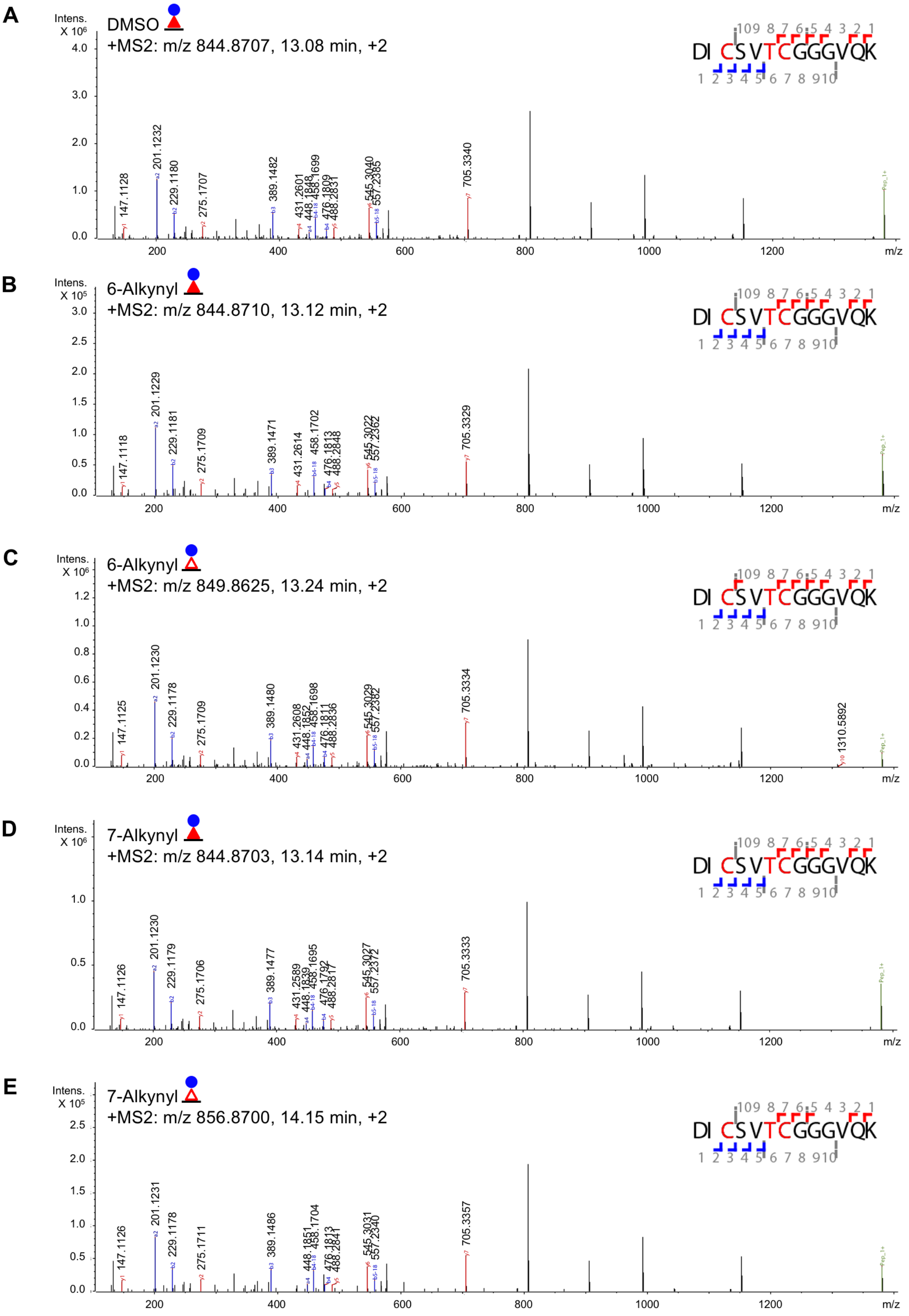

Supplemental Figure 4. Mass spectra corresponding to EICs in Supplemental Figure S3. MS2 spectra of glycoforms of the peptide DICSVTCGGGVQK from hTHBS1 TSR3 confirm the peptide assignment. (**A**) MS2 spectra of the peptide modified with Glc-Fuc disaccharide in sample treated with DMSO. (**B**) MS2 of the peptide modified with Glc-Fuc disaccharide in sample treated with peracetylated 6-Alk-Fuc. (**C**) MS2 spectra of the peptide modified with Glc-6-Alk-Fuc disaccharide in sample treated with peracetylated 6-Alk-Fuc. (**D**) MS2 spectra of the peptide modified with Glc-Fuc disaccharide in sample treated with peracetylated 7-Alk-Fuc. (**E**) MS2 of a peptide modified with glucose-7-Alk-Fuc disaccharide in sample treated with peracetylated 7-Alk-Fuc.

Supplemental Figure 5

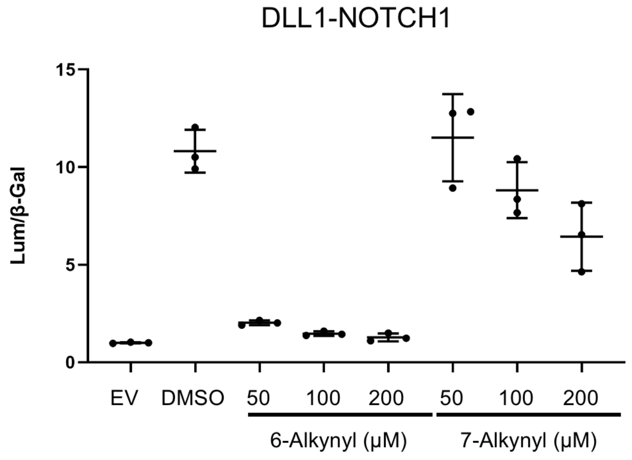

Supplemental Figure 5. 6-Alk-Fuc but not 7-Alk-Fuc inhibits DLL1-mediated Notch signaling. NIH3T3 cells expressing mNOTCH1 were co-cultured with L-cells expressing DLL1 to initiate Notch signaling. Cells were incubated with 50 μM, 100 μM or 200 μM 6-Alk-Fuc or 7-Alk-Fuc for 24 h before testing. Cells transfected with empty vector (EV) were used as a negative control for Notch signaling. Cells expressing mNOTCH1 and treated with DMSO were used as a positive control. All plots represent mean ± SD. Lum, luminescence; β-Gal, β-galactosidase.

Supplementary Figure 6

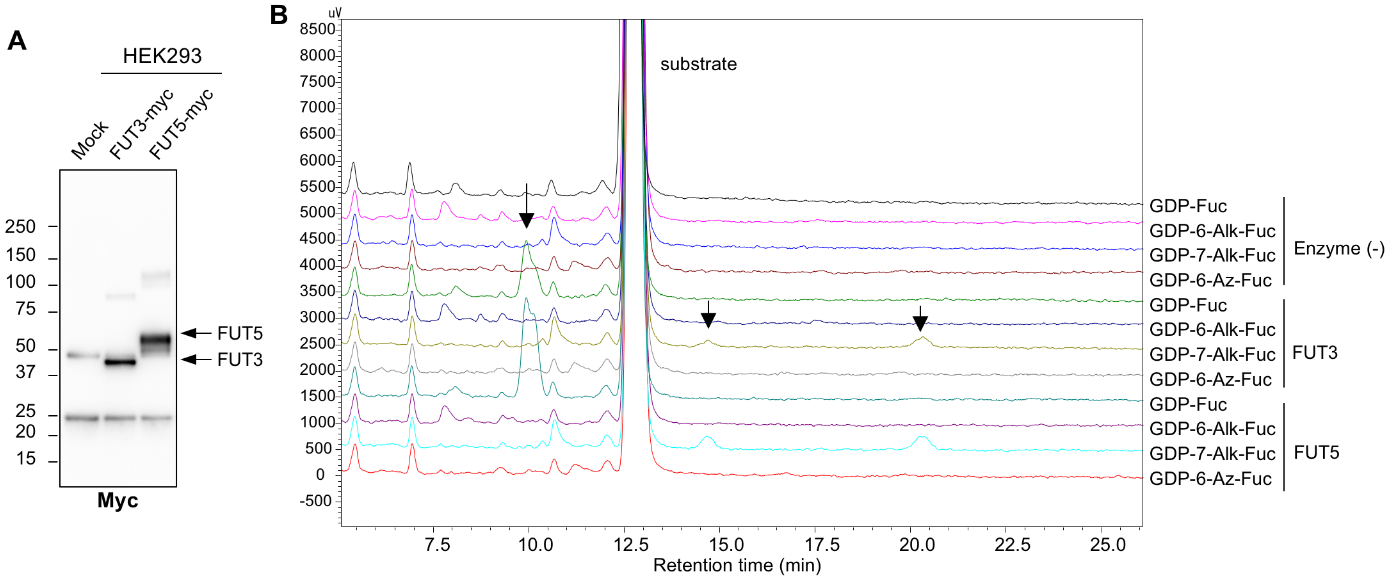

Supplementary Figure 6. In vitro activity assay of FUT3 and FUT5. (**A**) Full length myc-tagged human FUT3 and FUT5 were overexpressed in HEK293 cells, and immunopurified with anti-myc Ab and western blotted with the same Ab. (**B**) The purified enzymes were incubated with acceptor substrate (GGnGGnbi-PA) and donor substrate (GDP-Fuc or GDP-Fuc analog), and the reaction mixtures were analyzed by reversed-phase HPLC. Arrows indicate the products of enzymatic reactions.

Supplementary Figure 7

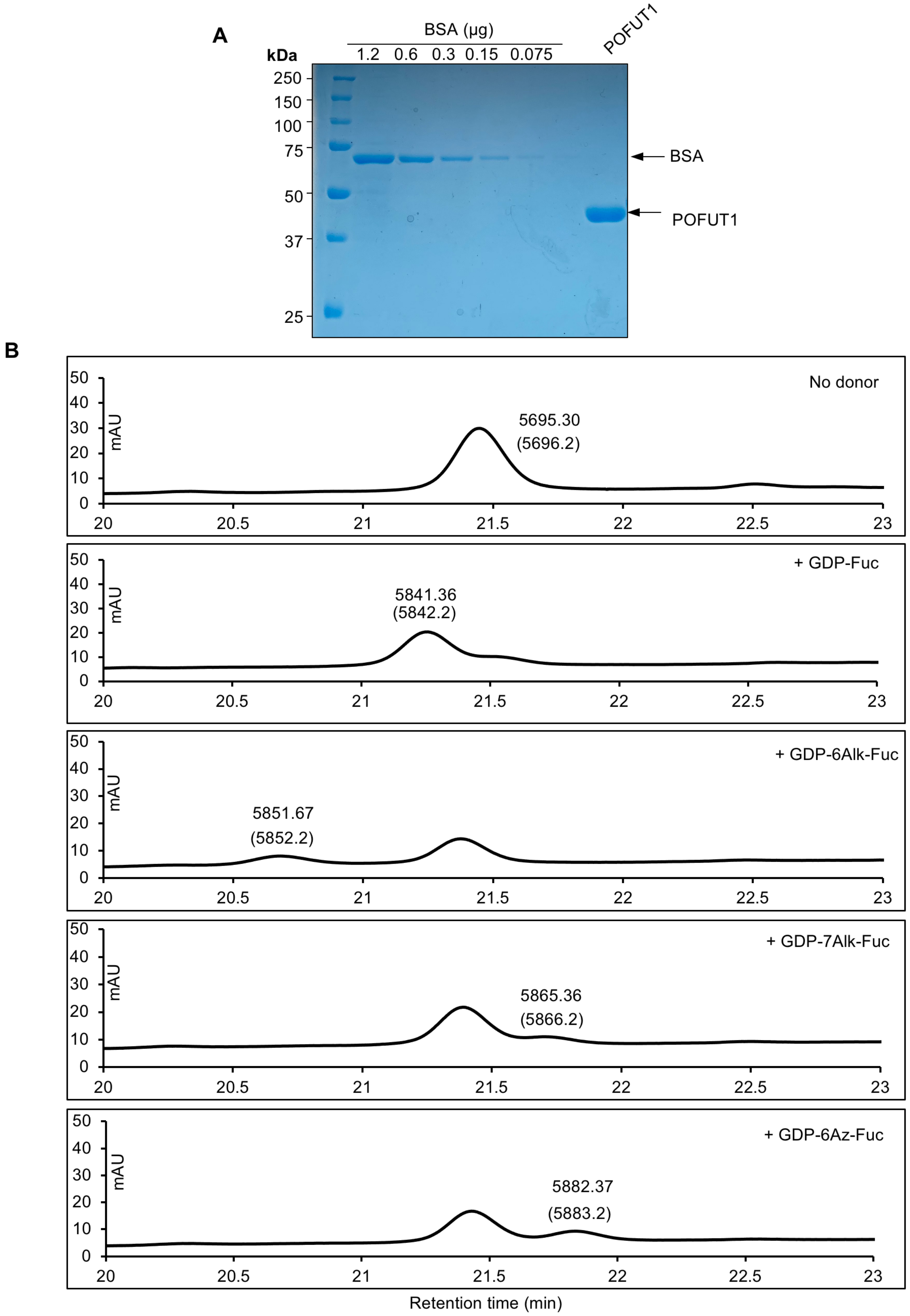

Supplementary Figure 7. In vitro activity assay of POFUT1. (**A**) His-tagged human POFUT1 lacking C-terminal RDEF sequence (POFUT1) proteins were expressed in HEK293T cells, purified from the medium by Ni-NTA agarose, and quantified by Coomassie brilliant blue staining. (**B**) The purified enzyme was incubated with acceptor substrate (human factor IX EGF1) and donor substrate (GDP-Fuc or GDP-Fuc analog), and the reaction mixtures were analyzed by reversed-phase HPLC. The elution profiles are shown, and determined masses (Da) are indicated. The predicted masses (Da) of proteins are indicated in parentheses.

Supplementary Figure 8

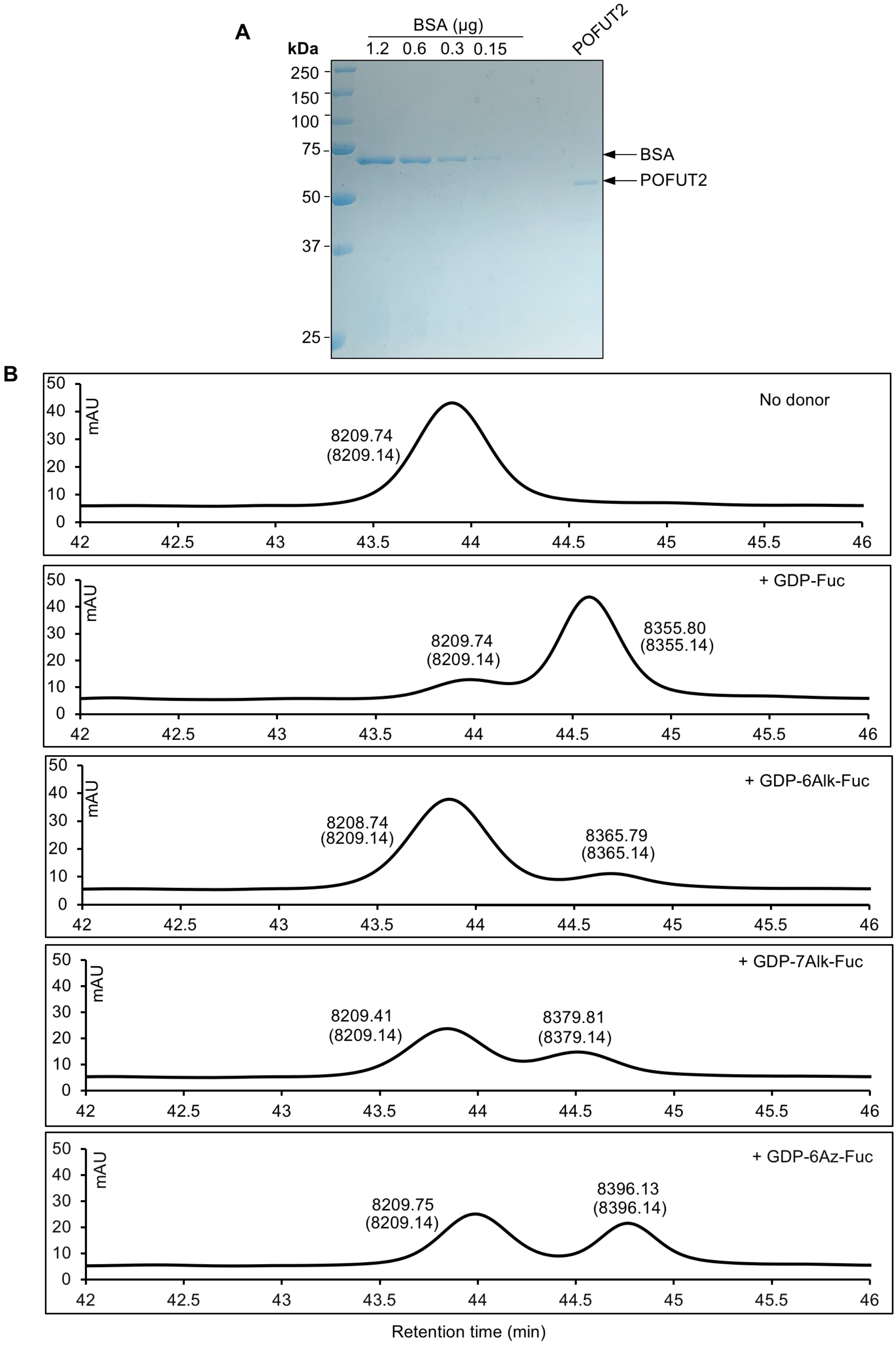

Supplementary Figure 8. In vitro activity assay POFUT2. (**A**) The flag-tagged mouse POFUT2 (POFUT2) was expressed in HEK293T cells and purified by DDDDK-tagged protein purification gel from cell lysates. POFUT2 enzyme was quantified by Coomassie brilliant blue staining . (**B**) The purified enzyme was incubated with acceptor substrate (TSP1-TSR3) and donor substrate (GDP-Fuc or GDP-Fuc analog), and the reaction mixtures were analyzed by reversed-phase HPLC. The elution profiles are shown, and determined masses (Da) are indicated. The predicted masses (Da) of proteins are indicated in parentheses.
